# Clinical impact of carbapenems in critically ill patients with valproic acid therapy: A propensity-matched analysis

**DOI:** 10.3389/fneur.2023.1069742

**Published:** 2023-03-09

**Authors:** Shu-Chen Hsiao, Wei-Hung Lai, I-Ling Chen, Fu-Yuan Shih

**Affiliations:** ^1^Department of Pharmacy, Kaohsiung Chang Gung Memorial Hospital, Kaohsiung, Taiwan; ^2^Department of Trauma Surgery, Chang Gung University College of Medicine, Kaohsiung Chang Gung Memorial Hospital, Kaohsiung, Taiwan; ^3^School of Pharmacy, Kaohsiung Medical University, Kaohsiung, Taiwan; ^4^Department of Neurosurgery, Chang Gung University College of Medicine, Kaohsiung Chang Gung Memorial Hospital, Kaohsiung, Taiwan

**Keywords:** carbapenem, valproic acid, drug-drug interaction, critical care outcome, status epilepticus, mortality, healthcare resource

## Abstract

**Background:**

Valproic acid (VPA) is one of the most widely used broad-spectrum antiepileptic drugs, and carbapenems (CBPs) remain the drug of choice for severe infection caused by multidrug-resistant bacteria in critically ill patients. The interaction between VPA and CBPs can lead to a rapid depletion of serum VPA level. This may then cause status epilepticus (SE), which is associated with significant mortality. However, the prognostic impact of drug interactions in critically ill patients remains an under-investigated issue.

**Objective:**

The aim of this study was to compare the prognosis of critically ill patients treated with VPA and concomitant CBPs or other broad-spectrum antibiotics.

**Methods:**

Adult patients admitted to a medical center intensive care unit between January 2007 and December 2017 who concomitantly received VPA and antibiotics were enrolled. The risk of reduced VPA serum concentration, seizures and SE, mortality rate, length of hospital stay (LOS), and healthcare expenditure after concomitant administration were analyzed after propensity score matching.

**Results:**

A total of 1,277 patients were included in the study, of whom 264 (20.7%) concomitantly received VPA and CBPs. After matching, the patients who received CBPs were associated with lower VPA serum concentration (15.8 vs. 60.8 mg/L; *p* < 0.0001), a higher risk of seizures (51.2 vs. 32.4%; adjusted odds ratio [aOR], 2.19; 95% CI, 1.48–3.24; *p* < 0.0001), higher risk of SE (13.6 vs. 4.7%; aOR, 3.20; 95% CI, 1.51–6.74; *p* = 0.0014), higher in-hospital mortality rate (33.8 vs. 24.9%; aOR, 1.57; 95% CI, 1.03–2.20; *p* = 0.036), longer LOS after concomitant therapy (41 vs. 30 days; *p* < 0.001), and increased healthcare expenditure (US$20,970 vs. US$12,848; *p* < 0.0001) than those who received other broad-spectrum antibiotics.

**Conclusion:**

The administration of CBPs in epileptic patients under VPA therapy was associated with lower VAP serum concentration, a higher risk of seizures and SE, mortality, longer LOS, and significant utilization of healthcare resources. Healthcare professionals should pay attention to the concomitant use of VPA and CBPs when treating patients with epilepsy. Further studies are warranted to investigate the reason for the poor outcomes and whether avoiding the co-administration of VPA and CBP can improve the outcomes of epileptic patients.

## 1. Introduction

Seizures are common neurological complications in the critically ill (1). The severity of seizures exists from a single seizure to status epilepticus, which is associated with high mortality (2) and longer length of hospital stay (LOS) (3). Seizures and status epilepticus (SE) may be due to a history of epilepsy or secondary insults such as acute stroke, traumatic brain injury, brain tumor, central nervous system infection, electrolytic and metabolic disorders, sepsis, medication withdrawal, drug toxicity, and organ failure (4). Antiepileptic drugs (AEDs) withdrawal or noncompliance is the common cause of SE (5, 6).

An international study reported that 70% of patients receive empirical or targeted antibiotic treatment in the intensive care unit (ICU) (7). Multiple classes of antibiotics are associated with symptomatic seizures and SE (8, 9). Therefore, when prescribing antibiotics to patients with epilepsy, some issues have to be considered, including whether they will adversely affect seizure control, precipitate seizures, and interact with concomitant AEDs (10).

Carbapenems (CBPs), such as imipenem/cilastatin, meropenem, ertapenem, and doripenem, have a broad antimicrobial spectrum and are used to treat severe and complicated bacterial infections (11). The interaction between CBPs and valproic acid (VPA), which is a broad-spectrum AED and widely used for acute and chronic seizures (12), may worsen seizure control and increase the risk of developing SE (13–19).

Infection (20, 21) and SE (2, 22) are associated with LOS, mortality, and cost in critically ill patients. However, the effect of the interaction between CBPs and VPA on the outcomes of critically ill patients remains uncertain. Therefore, this study aimed to use propensity score matching (23, 24) and compared the risk of seizures and SE, mortality rate, and healthcare resource utilization between critically ill patients treated with VPA and concomitant CBPs or other broad-spectrum antibiotics.

## 2. Materials and methods

### 2.1. Study population and data collection

Critically ill adult patients (age: 18–99 years) who were admitted to the ICUs at Kaohsiung Chang Gung Memorial Hospital between 2007 and 2017 and received VPA for a history of epileptic seizures were enrolled in this cohort study. Of these patients, those who concomitantly received antibiotics were divided into two groups: CBP group or other broad-spectrum antibiotics group (non-CBP group). The following carbapenem antibiotics were included in this study: imipenem/cilastatin, meropenem, ertapenem, and doripenem. We excluded patients who stayed in the ICU for <48 h, those who received VPA therapy for <48 h, and those who developed SE before concomitant administration. The study was approved (approval no. 201800716B0) by the Institutional Review Board of Chang Gung Medical Foundation, which waived the requirement for written informed consent.

Clinical information was retrieved and reviewed from the patient's medical records and the Chang Gung Research Database, which contains information on demographics, pharmacy dispensing, and clinical measures, including diagnosis, laboratory results, and healthcare expenditure. The collected variables included age, sex, etiology of epileptic seizures, AEDs used, Sequential Organ Failure Assessment (SOFA) score (25), Charlson comorbidity index score (26), hospital-acquired infection (27), comorbidities, VPA serum concentration, epileptic seizures, SE, in-hospital mortality, length of hospital stay (LOS), and healthcare expenditure. In addition, data on comorbidities including cerebrovascular disease, myocardial infarction, congestive heart failure, peripheral vascular disease, chronic pulmonary disease, liver disease, chronic kidney disease, diabetes mellitus, and malignant neoplasms were also recorded.

### 2.2. Outcome measures and definitions

The primary outcome was the risk of lower VPA serum concentrations and the risk of seizures and SE during concomitant administration. SE was defined according to the International League Against Epilepsy as seizures lasting more than 5 min or recurrent epileptic activity over a period of more than 5 min without regaining the pre-existing level of consciousness (28). The secondary outcomes included in-hospital mortality rate, LOS after concomitant administration, and healthcare expenditure.

The calculation of hospital expenditure included the official estimated cost per day of hospitalization in a standard ward and the ICU. This cost included all medical services, diagnostic tests, complementary examinations, therapeutic procedures, medications, and the materials needed during the patient's admission.

### 2.3. Statistical analysis

The patients' demographics, clinical characteristics, and outcomes are summarized using frequency and percentage for categorical variables and median and interquartile range for continuous variables. As appropriate, comparisons between groups were performed using the Pearson chi-square test and Wilcoxon rank-sum test. To overcome selection bias between the CBP and non-CBP groups, we performed propensity score matching by fitting a logistic regression with a greedy 8→ 1 digit-matching algorithm (29). Cases were initially matched to controls on 8 decimals of the propensity score. Those who were not matched on 8 decimals were matched using 7 decimals, and so forth down to a 1-decimal match. The CBP group of patients who remained unmatched at 1 decimal of the propensity score were excluded from the analysis. The covariates included in the propensity score model were those with a potential impact on the outcome: age, sex, etiology of epileptic seizures, AEDs used, SOFA score, Charlson comorbidity index score, hospital-acquired infection, and comorbidities. The Kaplan-Meier method was used to estimate the cumulative risk of developing SE after concomitant administration. All analyses were performed using SAS version 9.4 (SAS Institute, Cary NC, USA).

## 3. Results

### 3.1. Baseline characteristics

**Figure 1:**
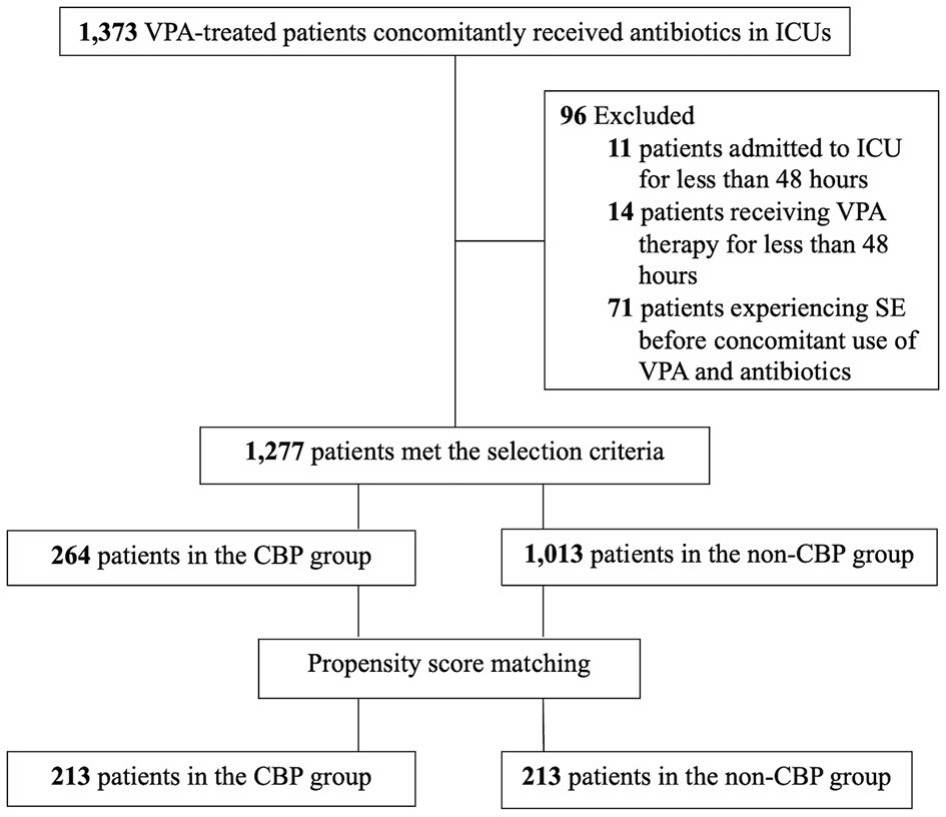
The enrollment flowchart of critically ill patients treated with VPA and concomitant antibiotics. CBP, carbapenem antibiotics; ICU, intensive care unit; SE, status epilepticus; VPA, valproic acid.

A total of 1,373 patients in ICUs concomitantly received VPA and antibiotics during the study period, of whom 96 were excluded (11 who stayed in the ICU for <48 h, 14 who received VPA therapy for <48 h, and 71 who developed SE before concomitant administration). The remaining 1,277 patients met the selection criteria and were enrolled, including 264 patients in the CBP group and 1,013 patients in the non-CBP group (Figure 1). The baseline characteristics of the included patients before matching are shown in Table 1. The median age of the patients was 66 years (range: 18–99 years), and the majority were male (61.2%). The most common etiology of epileptic seizures was post-stroke. Seven hundred and forty-two patients (58.1%) underwent monotherapy with VPA. The median SOFA score was 6, and the median Charlson comorbidity index score was 3. The most common comorbidity was cerebrovascular disease (57.1%), and 131 patients (10.3%) had hospital-acquired infections. The in-hospital mortality rate was 21.5%, the median length of LOS after concomitant administration was 31 days, and the median healthcare expenditure was US$6,764.

**Table 1:** Characteristics of VPA-treated patients with concomitant antibiotics use (*N* = 1,277).

**Characteristics**	**Value**
Age (years), median (IQR)	66 (54–77)
Sex, *n* (%)	
Male	782 (61.2)
Female	495 (38.8)
Etiology of epileptic seizures, *n* (%)	
Post-stroke	689 (54.0)
Post-traumatic	335 (26.2)
CNS infection	175 (13.7)
Brain tumor	125 (9.8)
Other	210 (16.4)
AEDs used, *n* (%)	
Monotherapy	742 (58.1)
Dual therapy	340 (26.6)
Polytherapy (≧3)	195 (15.3)
SOFA score, median (IQR)	6 (5–9)
Charlson comorbidity index, median (IQR)	3 (1–5)
Hospital-acquired infection, *n* (%)	131 (10.3)
Comorbidities, *n* (%)	
Cerebrovascular disease	729 (57.1)
Myocardial infarction	114 (8.9)
Congestive heart failure	199 (15.6)
Peripheral vascular disease	84 (6.6)
Chronic pulmonary disease	376 (29.4)
Liver disease	262 (20.5)
Chronic renal disease	297 (23.3)
Diabetes mellitus	478 (37.4)
Malignant neoplasms	276 (21.6)
Length of hospital stay after concomitant use of VPA and antibiotics (days), median (IQR)	31 (17–50)
In-hospital mortality, *n* (%)	275 (21.5)
Expenditure of healthcare, U.S. dollars, median (IQR)	6,764.0 (3,622.8–12,022.2)

AEDs, antiepileptic drugs; IQR, interquartile range; n, number; SOFA, Sequential Organ Failure Assessment; VPA, valproic acid.

Before propensity score matching, the characteristics varied considerably between the two groups (Table 2). The CBP group was older and had more post-stroke seizures (*p* = 0.015), more post-traumatic seizures (*p* = 0.010), a higher SOFA score (*p* < 0.001), a higher Charlson comorbidity index score (*p* < 0.001), and a higher hospital-acquired infection rate (*p* < 0.001). In addition, the non-CBP group had higher rates of comorbidities, including congestive heart failure (*p* = 0.014) and chronic renal disease (*p* < 0.001). After propensity score matching, 213 patients were assigned to each group. There were no significant differences between the two matched groups.

**Table 2:** Characteristics of VPA-treated patients with concomitant antibiotics use before and after propensity score matching.

**Variables**	**Before propensity score matching (*****n*** = **1,277)**			**After propensity score matching (*****n*** = **426)**		
	**CBP group (*****n*** = **264)**	**non-CBP group (*****n*** = **1,013)**	***P***	**CBP group (*****n*** = **213)**	**non-CBP group (*****n*** = **213)**	***P***
Age (years), median (IQR)	68 (58–79)	65 (52–77)	0.011	68 (58–78)	69 (57–77)	0.543
Sex					
Male, *n* (%)	168 (63.6)	614 (60.6)	0.369	133 (62.4)	140 (65.7)	0.480
Female, *n* (%)	96 (36.4)	399 (39.4)		80 (37.6)	73 (34.3)	
Etiology of epileptic seizures, *n* (%)					
Post-stroke	160 (60.6)	529 (52.2)	0.015	142 (66.7)	140 (65.7)	0.838
Post-traumatic	53 (20.1)	282 (27.8)	0.010	42 (19.7)	46 (21.6)	0.632
CNS infection	36 (13.6)	139 (13.7)	0.945	23 (10.8)	23 (10.8)	1.000
Brain tumor	28 (10.6)	97 (9.6)	0.636	30 (14.1)	34 (16.0)	0.588
Other	50 (18.9)	160 (15.8)	0.222	36 (16.9)	36 (16.9)	1.000
AEDs used, *n* (%)					
Monotherapy	168 (63.6)	574 (56.7)	0.078	132 (62.0)	137 (64.3)	0.443
Dual therapy	66 (25.0)	274 (27.0)		55 (25.8)	58 (27.2)	
Polytherapy (≧3)	30 (11.4)	165 (16.3)		26 (12.2)	18 (8.5)	
SOFA score, median (IQR)	8 (5–11)	6 (5-8)	<0.001	8 (5–10)	8 (6–10)	0.818
Charlson comorbidity index, median (IQR)	4 (2-6)	3 (1–5)	<0.001	4 (2–6)	4 (2–6)	0.810
Hospital-acquired infection, *n* (%)	51 (19.3)	80 (7.9)	<0.001	36 (16.6)	34 (16.0)	0.794
Comorbidities, *n* (%)					
Cerebrovascular disease	164 (62.1)	565 (55.8)	0.064	133 (62.4)	130 (61.0)	0.765
Myocardial infarction	17 (6.4)	97 (9.6)	0.112	9 (4.2)	16 (7.5)	0.149
Congestive heart failure	145 (14.3)	54 (20.5)	0.014	44 (20.7)	47 (22.1)	0.772
Peripheral vascular disease	65 (6.4)	19 (7.2)	0.649	17 (8.0)	16 (7.5)	0.856
Chronic pulmonary disease	87 (33)	289 (28.5)	0.160	69 (32.4)	74 (34.7)	0.608
Liver disease	64 (24.2)	198 (19.6)	0.092	49 (23.0)	49 (23.0)	1.000
Chronic renal disease	83 (31.4)	214 (21.1)	<0.001	66 (31.0)	79 (37.1)	0184
Diabetes mellitus	376 (37.1)	102 (38.6)	0.710	77 (36.2)	83 (39.0)	0.777
Malignant neoplasms	63 (23.9)	213 (21)	0.319	48 (22.5)	54 (25.4)	0.496

AEDs, antiepileptic drugs; CBP, carbapenem antibiotics; CNS, central nerve system; IQR, interquartile range; n, number; SOFA, Sequential Organ Failure Assessment; VPA, valproic acid.

### 3.2. Outcomes associated with co-administration of VPA and antibiotics

**Figure 2:**
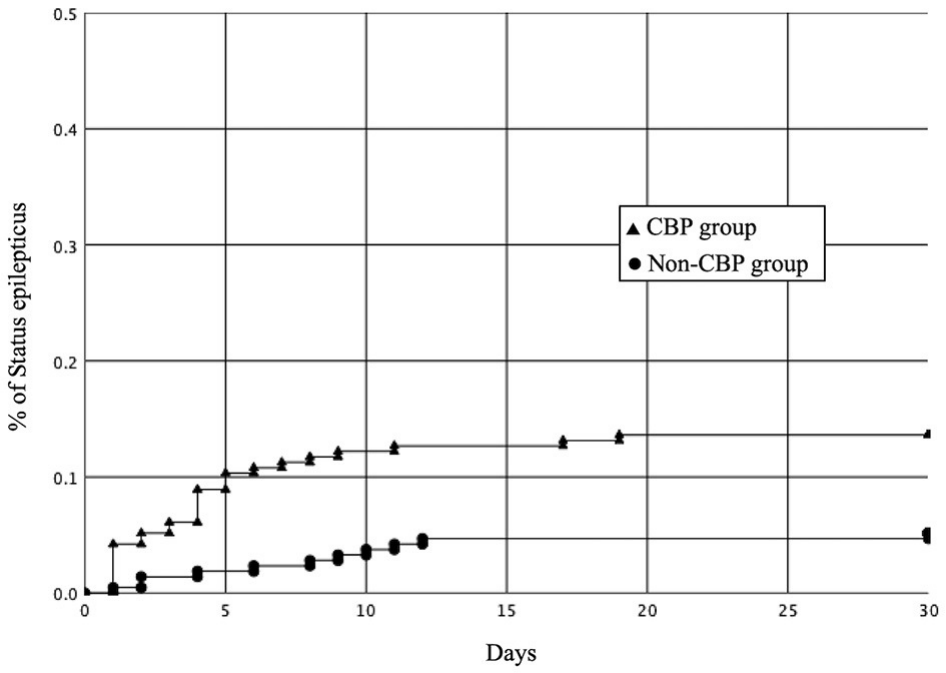
Kaplan-Meier curves showing the rates of status epilepticus in the CBP and non-CBP groups after the concomitant administration (log-rank *P* = 0.001). CBP, carbapenem antibiotics.

We then investigated the outcomes of the patients in the CBP group and non-CBP group (Table 3). The CBP group had a lower median VPA serum concentration (15.8 versus 60.8 mg/L; *p* < 0.0001). One hundred and seventy-eight patients with epileptic seizures had 124 electroencephalography (EEG) records. Regarding EEG patterns, 60 patients had delta activity, 3 patients had inter-ictal spikes, 44 patients had electric seizures, 4 patients had lateralized periodic discharge, 12 patients had status epilepticus, and one had normal patterns. Epileptic seizures occurred in 178 (41.8%) of the patients overall, including 109 (51.2%) in the CBP group and 69 (32.4%) in the non-CBP group (adjusted odds ratio [aOR], 2.19; 95% CI, 1.48–3.24; *p* < 0.001). SE occurred in 39 (9.2%) of the patients overall, including 29 (13.6%) in the CBP group and 10 (4.7%) in the non-CBP group (aOR, 3.20; 95% CI, 1.51–6.74; *p* = 0.001). There were four patients with non-convulsive seizures. After concomitant administration, the epileptic seizure and SE rates were higher in the CBP group. The Kaplan-Meier curve showing the relationship between the duration of concomitant administration and SE is shown in Figure 2. The 14-day SE rate was 12.7% in the CBP group and 4.7% in the non-CBP group (log-rank *p* = 0.002).

**Table 3:** Comparison of clinical outcomes in propensity-matched groups.

**Clinical outcomes**	**CBP group (*n* = 213)**	**non-CBP group (*n* = 213)**	**aOR (95% CI)*; p*^‡^**
VPA serum concentration (mg/L), median (IQR)	15.8 (7.5-24.9); (*n* = 192)	60.8 (48.3-79.0); (*n* = 191)	*p* < 0.0001^*^
Epileptic seizures during concomitant administration, *n* (%)	109 (51.2)	69 (32.4)	2.19 (1.48–3.24); *p* < 0.0001
Status epilepticus during concomitant administration, *n* (%)	29 (13.6)	10 (4.7)	3.20 (1.51–6.74); *p* = 0.0014
In-hospital mortality rate, *n* (%)	72 (33.8)	53 (24.9)	1.57 (1.03–2.20); *p* = 0.036
Length of hospital stay after concomitant administration (days), median (IQR)	41 (23–66)	30 (26–48)	*p* < 0.001^*^
Expenditure of healthcare, U.S. dollars, median (IQR)	20,970 (13,321.4-34,549.1)	12,848 (7,314.4-22,831.6)	*p* < 0.0001^*^

aOR, adjusted odds ratio; CBP, carbapenem antibiotics; CI, confidence interval; IQR, interquartile range; *n*, number;

‡ *P-*value is reported for Chi-square test unless otherwise stated;

* *P*-value is reported for Wilcoxon test; VPA, valproic acid.

We also investigated the two groups' mortality, LOS, and healthcare expenditure. The CBP group was associated with a higher in-hospital mortality rate (33.8% versus 24.9%, aOR, 1.57; 95% CI, 1.03–2.20; *p* = 0.036), longer LOS after concomitant administration (41 vs. 30 days; *p* < 0.001), and increased healthcare expenditure (US$20,970 versus US$12,848; *p* < 0.001) compared to the non-CBP group (Table 3).

## 4. Discussion

### 4.1. Outcomes associated with co-administration of VPA and antibiotics

In this retrospective propensity-matched cohort study, 41.8% of the critically ill enrolled patients had epileptic seizures, and 9.2% had SE during the concomitant administration of VPA and antibiotics. In addition, the co-administration of VPA and CBP was associated with lower VAP serum concentration, a higher risk of epileptic seizures and SE, higher mortality rate, longer LOS, and higher healthcare expenditure compared to the concomitant administration of VPA and other broad-spectrum antibiotics.

The prevalence of epileptic seizures in critically ill patients has been reported to range from 8 to 34% in previous studies (30–38). Moreover, in critically ill patients with a history of epileptic seizures, the epileptic seizure rate has been reported to range from 33 to 61% (32–34). In the current study, 41.8% of the patients had epileptic seizures, which is consistent with previous studies.

Low AED levels in patients with epilepsy are one of the most common etiologies of epileptic seizures and SE (32, 34, 39, 40). Many etiologies can cause epileptic seizures in the ICU, including alcohol withdrawal, stroke, anoxic brain injury, central nervous system infection, head trauma, sepsis, metabolic disorders, and other acute drug toxicity or withdrawal (41). Although CBPs have been associated with a higher risk of epileptic seizures than non-CBP antibiotics, the absolute risk of epileptic seizures with CBPs is low (42). In our study, all patients had a history of epileptic seizures and underwent AED therapy before ICU admission. After propensity score matching, the CBP group had a higher risk of epileptic seizures than the non-CBP group. Pre-existing epilepsy and AED withdrawal are the most common etiologies associated with epileptic seizures in critically ill patients (32, 34, 39). The co-administration of VPA and CBPs has been shown to be similar to the abrupt withdrawal of VPA and may cause serious rebound epileptic seizures (13–19). The VAP median serum concentration in the CBP group was lower than in the non-CBP group (15.8 vs. 60.8 mg/L; *p* < 0.0001). Several mechanisms have been proposed for the drug-drug interactions between VPA and CBPs, including the inhibition of intestinal VPA absorption (43), interruption of enterohepatic circulation of VPA (44), inhibition of VPA efflux from erythrocytes (45), increased VAP glucuronidation (46), inhibition of hydrolysis of VPA glucuronide (VPA-G) to VPA (47), and elevation in urinary excretion of VPA-G (48). Although the precise mechanisms remain unclear, one of the generally recognized explanations is that CBPs inhibit the hydrolysis of VPA-G to VPA, resulting in the rapid hepatic clearance of VPA-G and subsequent decline in plasma VPA concentration, which in turn is associated with a higher risk of epileptic seizures and SE. Concomitant administration with VPA and CBPs has also been associated with an increased number of epileptic seizures and epileptic activity on EEG (15). Acylpeptide hydrolase (APEH) is the key enzyme responsible for VPA-G hydrolysis (49), and CBPs have been associated with the irreversible inhibition of APEH (50). Following the discontinuation of CBP treatment, the effect on VAP has been noted to last for several days (13, 15, 16). This may be why there was a higher 14-day SE rate in the CBP group (12.7%) than in the non-CBP group (4.7%).

The mortality rate in critical care units has been associated with multiple factors, including age (51), infection (52), comorbidities (53), and SE (2). Before matching, the CBP group was older and had a higher SOFA score, higher Charlson comorbidity index score, and higher hospital-acquired infection rate. SOFA score and Charlson comorbidity index are used to predict infection-related mortality (54) and in-hospital mortality (53) in the ICU. After matching, the SOFA score was 8 in both groups. The reported mortality rate of critically ill patients with infection is around 20% (54). However, the mortality rate was 33.8% in our CBP group. The all-cause in-hospital mortality rate in patients receiving coadministration of VAP and CBP has been reported to range from 42.9 to 64% (13, 15). The higher rate than in our study may be due to the small sample size and comorbidities in the previous studies. SE is associated with increased mortality in critically ill patients (2, 55). In our study, the mortality rate in the patients who developed SE was 43.6% (17 of 39). This result is consistent with the findings in previous studies, in which the in-hospital mortality rate of SE ranged from 38 to 55% (56, 57). Infection after SE is frequent and associated with higher mortality (58). However, no previous study has focused on SE after infection. In the present study, we found that the concomitant administration of VPA and CBPs was a risk factor compared to increased mortality (33.8 vs. 24.9%), which may be due to the increased risk of SE in the CBP group.

SE has been associated with high healthcare expenditure in previous studies (3, 55, 59) due to lengthy hospitalization and possible sequelae (3). In previous studies from the US and Germany, the median inpatient costs of SE were US$18,834 and €4,702 per admission (55, 60). In our study, the median inpatient cost was higher for the patients with SE than those without SE (US$21,272 vs. US$16,304, *p* = 0.011). Several studies have shown that higher severity of illness corresponds to higher ICU costs (61, 62) and that 85% to 90% of ICU and post-ICU hospital costs are due to LOS (61). A longer LOS was noted after the concomitant administration of VAP and CBPs in the present study. Spriet et al. reported a median LOS of 46 days in patients who received concomitant administration with VAP and CBPs (15), comparable with our CBP group (41 days). In our study, the median LOS in the patients with SE was 7 days longer than in those without SE, however, the difference was not significant (42 vs. 35 days, *p* = 0.130). The results of the present study underscore the importance of decreasing SE by avoiding the concomitant use of VAP and CBP. Therefore, concomitant administration with VAP and CBPs may increase the LOS and healthcare expenditure.

### 4.2. Strengths and limitations

To the best of our knowledge, this is the first and largest study to investigate concomitant administration with VAP and CBPs in critically ill patients in the ICU. Using propensity score matching, the CBP group were associated with worse clinical outcomes than the non-CBP group. Our findings are important and strengthen the evidence for clinical decision-making when facing critically ill patients who require the concomitant use of AEDs with antibiotics. In addition, our study confirms and highlights that SE is associated with high mortality and significant utilization of healthcare resources.

In the present study, we used propensity score matching to evaluate the relationship between VPA-CBP interactions and critical care outcomes as it can reduce confounding by balancing the observed covariates at each particular value of the propensity score (63). After matching, all selected potential confounding factors were sufficiently balanced between the two matched groups. This is similar to randomization procedures used in clinical trials, as on average the distribution of covariates was balanced between the CBP and non-CBP groups, which strengthens causal inference and thus improves the methodological quality of this observational study.

This study has several limitations. First, this was an observational and not a randomized study, and so we cannot exclude the possibility of unmeasured confounding. For example, not every patient with seizures had EEG recordings. Interventions which may have reduced morbidity and mortality such as treatment of refractory SE with general anesthesia or AED combination therapy (64, 65). Second, the use of propensity score matching limited the sample size to patients who could be matched. Third, this was a single-center study, and results may differ in other setting and other populations.

## 5. Conclusions

Our results provide the strongest observational evidence to date of the effect of concomitant administration with VAP and CBPs in critically ill ICU patients. Our results highlight that the CBPs should be avoided prescribing to patients with epilepsy undergoing VPA therapy in ICU. If patients with VAP need CBP therapy, we should monitor seizures closely and manage seizures carefully. Changing AED will be better management than increasing the VPA dosage. Further studies are warranted to investigate the reason for the poor outcomes and whether avoiding the co-administration of VPA and CBP can improve the outcomes of epileptic patients.

## Data availability statement

The raw data supporting the conclusions of this article will be made available by the authors, without undue reservation.

## Ethics statement

The studies involving human participants were reviewed and approved by Institutional Review Board of Chang Gung Medical Foundation. Written informed consent for participation was not required for this study in accordance with the national legislation and the institutional requirements.

## Author contributions

S-CH, I-LC, and F-YS designed the study. I-LC and S-CH built the database. S-CH wrote the first version of the manuscript. S-CH and F-YS performed the first revision of the manuscript. I-LC and F-YS performed the statistical analyses. All authors contributed to the interpretation of the data, revised the manuscript critically, gave final approval of the version to be published, and read and approved the final manuscript.
